# Perspectives in adhesion prevention in gynaecological surgery

**DOI:** 10.52054/FVVO.15.4.108

**Published:** 2023-12-13

**Authors:** L.A. Torres-de la Roche, U Catena, T.J. Clark, R Devassy, N Leyland, R.L. De Wilde

**Affiliations:** on behalf of the Special Interest Group Adhesions Research of the European Society for Gynaecological Endoscopy (ESGE); University Hospital for Gynaecology, Pius-Hospital, University Medicine Oldenburg, 26121 Oldenburg, Germany; Division of Gynaecologic Oncology, Fondazione Policlinico Universitario A. Gemelli IRCCS, 00168 Rome, Italy; Department of Obstetrics and Gynaecology, Birmingham Women’s Hospital, Mindelsohn Way, Birmingham B15 2TG, UK; Institute of Metabolism and Systems Research, University of Birmingham, Birmingham B15 2TT, United Kingdom; Department of Obstetrics and Gynaecology, McMaster University, Hamilton, ON, L8S 4K1, Canada

**Keywords:** Adhesion prevention, adhesion barriers, haemostats, surgical technique

## Abstract

Adhesions are a frequent, clinically relevant, and often costly complication of surgery that can develop in any body location regardless of the type of surgical procedure. Adhesions result from surgical trauma inducing inflammatory and coagulation processes and to date cannot be entirely prevented. However, the extent of adhesion formation can be reduced by using good surgical technique and the use of anti-inflammatory drugs, haemostats, and barrier agents. Strategies are needed in the short-, medium- and longer-term to improve the prevention of adhesions. In the short-term, efforts are needed to increase the awareness amongst surgeons and patients about the potential risks and burden of surgically induced adhesions. To aid this in the medium- term, a risk score to identify patients at high risk of adhesion formation is being developed and validated. Furthermore, available potentially preventive measures need to be highlighted. Both clinical and health economic evaluations need to be undertaken to support the broad adoption of such measures. In the longer- term, a greater understanding of the pathogenic processes leading to the formation of adhesions is needed to help identify effective, future treatments to reliably prevent adhesions from forming and lyse existing ones.

## Introduction

Postoperative adhesions are a common complication occurring in up to 95% of patients regardless of body location and surgical procedure (Herrmann et al., 2020; Lauder et al., 2010). Amongst gynaecological procedures, endometriosis surgery (Mais et al., 1995), ovarian cystectomy (Keckstein et al., 1996), myomectomy (Herrmann et al., 2020), and oncological surgery (Fortin et al., 2015) are considered especially adhesiogenic, even when using minimally invasive techniques.

 Adhesions frequently lead to clinically relevant symptoms such as chronic pelvic pain (Ten Broek et al., 2013) and often costly complications (Krielen et al., 2020; Okabayashi et al., 2014; Stommel et al., 2018). A link between female subfertility and post- operative adhesions has frequently been postulated (Cates et al., 1985; Milingos et al.,2000; Ten Broek et al., 2013; Trimbos-Kemper et al., 1985) but the causal role of adhesions remains controversial (Herrmann et al., 2020). Moreover, almost all cases of small-bowel obstructions are adhesion-related (Ellis, 1971; Ellis, 1982; Herrmann et al., 2020; Menzies, 1993; Ten Broek et al., 2013).

### Mechanisms of adhesion formation

Adhesions result from inflammatory damage or surgical trauma to the peritoneal mesothelial layer (Fortin et al., 2015). They take different forms ranging from thin films of connective tissue to thick fibrous strands with their own vascularisation and nervous tissue (Fatehi Hassanabad et al., 2021). They are thus considered to be far more vital structures than just scar tissue. Their formation involves three core processes: (i) tissue hypoxia as a result of tissue injury, (ii) an altered coagulation cascade and inhibited fibrinolysis, resulting in the conversion of temporary fibrin bridges to dense, mature fibrous strands, and (iii) inflammatory processes, which regulate haemostasis and increase fibrin deposition (Fatehi Hassanabad et al., 2021, Fortin et al., 2015). An imbalance of inflammatory response, cellular migration, proliferation, angiogenesis, and tissue remodelling thus shifts the regular wound healing processes towards adhesion formation (Fatehi Hassanabad et al., 2021; Fortin et al., 2015). Exudation, and platelet and fibrin deposition occur within a few minutes after surgical trauma. Coverage of the denuded area by tissue repair cells takes minutes to several hours. Epithelial repair occurs between day 1 and day 3. In cases of delayed repair through local inflammation, fibroblast growth starts on day 3 and angiogenesis on day 5. Overall, bioactive mediators of post-surgical adhesions have been found for up to 7 days after surgery (Torres-De La Roche et al., 2019; Koninckx et al., 2016; Fatehi Hassanabad et al., 2021). There are no publications specifying the locations of adhesion formation in abdominal gynaecological surgery, other than at the site of iatrogenic trauma, and eventually concomitant at other places.

### Adhesion prevention - Current state-of-the-art

The need for surgical adhesiolysis represents an important healthcare burden (Sikirica et al., 2011) and remains unsustainable due to a high risk of adhesion reformation (Tittel et al., 2001). Surgical adhesiolysis is thus unsuitable as a standard procedure to overcome adhesion-related morbidity. The goal should therefore be to prevent adhesions from forming whenever possible. There are pharmacological and non-pharmacological agents helping to reduce the extent of adhesion formation (De Wilde et al., 2022).

Pharmacological agents are generally used to prevent inflammation (ibuprofen, celecoxib, resveratrol), reduce scarring and fibrosis (mitomycin C, pirfenidone), and to reduce blood clotting (heparin) (De Wilde et al., 2022).

Non-pharmacological agents include barriers and fluid agents, which are intended to separate abdominal and/or pelvic structures from injured tissues for a certain period of time to reduce the risk of post-operative formation of a fibrin matrix (Herrmann et al., 2020). Fluid agents include gels and hydroflotation agents. Anti- adhesive gels are often based on derivatives of hyaluronic acid or polyethylene glycol (PEG), while hydroflotation agents include high molecular weight glucose polymers or polysaccharide- containing solutions (Ahmad et al., 2020b). Barrier agents include oxidised regenerated cellulose, polytetrafluoroethylene, sodium hyaluronate with carboxymethylcellulose, and fibrin or collagen sheets (Ahmad et al., 2020a). A topic specifically related to laparoscopy is the ability to introduce adhesion barriers and place them effectively (Zhao et al., 2021). Instruments to facilitate this, have been developed and refinement should be achieved based on the feedback by surgeons (Kojima et al., 2020).

## Perspective

To improve the prevention and treatment of adhesions, there are important steps to be taken in both the short, medium, and longer-term. The immediately feasible steps encompass efforts to raise awareness among surgeons and patients about the importance, burden, and currently available preventive measures. Medium-range efforts comprise the development and validation of a risk score to identify patients at high risk of adhesion formation and allow the initiation of preventive measures. To ensure broad adoption of effective and cost-effective preventative measures, both clinical and health economic evaluations are necessary. In the longer-term, research needs to be pursued to fully understand the complexity of the pathogenic processes leading to the formation of adhesions and to use the resulting knowledge to find adequate treatments to prevent adhesions from forming and heal existing ones.

### Surgeon training and awareness

Surgeons need to be cognisant about the potential and importance of surgically induced adhesions. This includes their prevalence, risk factors, prevention methods, and treatment. Medicolegal implications regarding complications arising from post-operative adhesions need to be considered. It is therefore important that patients are fully counselled prior to surgery about the risk of adhesion formation and the possible consequences (De Wilde et al., 2022). This is especially important for potentially highly adhesiogenic surgical procedures. These include adnexal surgery such as ovarian cystectomy, excision of deep infiltrating endometriosis (DIE), hysterectomy in patients with DIE, myomectomy, and oncological procedures. It was initially thought that minimally invasive surgical procedures would lead to less trauma, fewer post-injury repair processes being activated and thus a lower risk of adhesion formation. In the 2020 update of the Surgical and Clinical Adhesions Research (SCAR) study, laparoscopic index surgery in the abdominopelvic cavity was associated with significantly fewer adhesion- related hospital readmissions than open surgery in the 5-year follow-up period (1.7% versus 4.3% directly adhesion-related, 16.0% versus 18.2% possibly related; p<0.005). In the subgroup analysis, however, this difference between open and laparoscopic surgery could not be confirmed for women undergoing surgery of the reproductive tract (Krielen et al. 2020). Hysteroscopic myoma resection was associated with intra-uterine adhesions in up to 78% of patients, depending on the number of myoma removed and the location of surgery (Zhang et al.2023). For robotic surgery, no further reduction in the incidence of adhesion formation compared to conventional laparoscopy has been reported, but in a recent systematic review and meta-analysis it was associated with a reduced risk of conversions (odds ratio [OR] 1.53, 95% CI 1.12–2.10, p=0.007) in patients in whom adhesions were the reason for conversion (Milone et al. 2022). Overall, however, studies and systematic reviews yielded conflicting results with regards to the differences in the rates of adhesion formation by surgical technique and the question has not yet been conclusively answered (Fatehi Hassanabad et al., 2021).

A surgical technique that is as least traumatic as possible should be adopted by every surgeon in every abdomino-pelvic surgical procedure (Herrmann et al., 2020) (Table I). This involves minimisation of the total length of the incision(s), careful consideration of the type and number of knots and suture material used, the avoidance of protruding wounds, the reduction of the pressure and duration of pneumoperitoneum in laparoscopy, meticulous haemostasis, and other important precautions outlined by the European Society for Gynaecological Endoscopy (De Wilde et al., 2014; De Wilde and Trew, 2007). To avoid risks associated with bleeding complications, fast-acting adjunct tools that work consistently across a range of bleeds should be considered (Slezak et al., 2020). Importantly, extensive electrocoagulation in the ovary should be avoided during endometrioma surgery due to a risk of subsequent infertility associated with “overheating” the ovary (Saridogan et al., 2017). Alternative approaches including mechanical haemostats (plant starch, gelatine, collagen, oxidised regenerated cellulose, polysaccharide spheres, mineral powders), active thrombin-based haemostats, flowable products (combinations of gelatine + thrombin), fibrin sealants (human plasma ± thrombin and collagen) might thus be preferred (Chung et al., 2021). Surgeon-validated scales for assessment of intraoperative bleeding severity have been realised in other fields (Lewis et al., 2017, Sciubba et al., 2022).

**Table I:** Risk factors and preventive factors in the formation of adhesions.

Risk factors	
	Mechanical trauma
	Inflammation
	Hypoxia
	Oxidative stress, reactive oxygen species
	Desiccation
	Presence of blood
	Sutures
	Infection
	Necrotic tissue
Preventive measures and factors	
	Gentle tissue handling
	Short duration of surgery
	Addition of more than 5% N_2_O to the CO_2_ pneumoperitoneum
	Cooling the abdomen to 30°C
	Anti-desiccation measures (heated humidified gases, Ringer’s lactate)
	Meticulous haemostasis
	No debris
	Avoid absorbable sutures

Source: Torres-De La Roche et al., 2019; Koninckx et al., 2016)

Systematic initiatives to improve surgical techniques, foster peer training, and engage in reciprocal and systematically documented peer-to- peer review visits could be used to quantify effects of adopted measures and improve outcomes. Such initiatives have, for example, been conducted in the framework of lung cancer diagnosis and treatment in the United Kingdom (Russell et al., 2014).

### Patient awareness and support

In recent years, patient groups concerned with adhesion have been forming. These groups will benefit from proactive engagement with professional surgeon societies and from the provision of evidence-based materials and events (Torres-De La Roche et al., 2019). Such activities could be used to raise awareness among patients but also to manage their expectations with respect to the benefits and risks of currently available methods and the clinical situations where they are beneficial (Lier et al., 2021; Lundorff et al., 2015).

### Need for a clinically validated risk score

There is a need to generate more evidence on predisposing patient-specific risk factors. A comprehensive literature review conducted in 2015 to identify predisposing factors to post-operative adhesion development (Fortin et al., 2015) discerned direct adhesiogenic risk factors and indirect ones that predispose individuals to an increased risk of fibrosis. Factors identified by Fortin et al. (2015) to directly increase the risk of adhesion formation are genetic polymorphisms, oestrogen exposure, and endometriosis. Indirect risk factors that disrupt the coagulation-fibrinolysis balance toward increased fibrosis are genetic polymorphisms, diabetes mellitus, metabolic syndrome, hyperglycaemia, obesity, depression, binge alcohol consumption, anti-Parkinsonian medications, oral hormone therapy, pregnancy and cancer.

A retrospective analysis of the Scottish Medical Record Linkage Database found a higher risk of adhesion-related readmission at a younger age, when malignancy was the indication for surgery, in case of intra-abdominal infection or previous radiotherapy, following application of a mesh, and with concomitant inflammatory bowel disease. Transvaginal surgery reduced the risk of adhesion-related complications as compared to laparoscopic or open surgeries (Toneman et al., 2023). Knowledge of these potential predisposing factors helps to identify high-risk patients who might benefit from adhesion prevention. Therefore, nomograms developed based on the analysis of the Scottish Medical Record Linkage Database help to visualise risk of readmission or reoperation due to adhesions (Toneman et al., 2023).

Based on the knowledge of pathophysiological, patient-level, and technique-related risk factors, a clinical adhesion score (CLAS) was developed and submitted to a two-step pilot study (Lier et al., 2021). The CLAS enables a complete and weighted evaluation of the consequences of adhesion- related complications following abdominal and pelvic surgery over a minimum of 24 months of follow-up with a recommendation of 36 months. Although first steps towards validation of the CLAS using retrospective data have been made, any standardisation in the form of a risk score will have to be submitted to a formal validation in a prospective clinical trial (De Wilde et al., 2022; Lier et al., 2021). Adhesion-related complications can occur over the course of several years after the index surgery, a stringently designed randomised, controlled trial will need to recruit a very large number of patients and will probably not be realistically feasible. A reasonable approximation could be justified by the fact that most hospital readmissions due to adhesions appear to occur within the first two years after surgery (Ellis et al., 1999; Krielen et al., 2020; Ten Broek et al., 2013).

Alternatively, existing registries could be adapted in order to provide readily accessible information on readmissions and other relevant indicators.

### Health economic evaluation

Any standardised intervention must also be economically justifiable. Evidence on the cost effectiveness and patient impact – i.e., clinical impact versus number of patients treated – must therefore be generated in order to support reimbursement and general uptake in clinical practice. This is especially important for public health care systems which conduct stringent health technology assessments to support reimbursement decisions. The goal is to establish an evidence- based treatment standard and to demonstrate a financial benefit of adhesion prevention. A recent study found direct health care costs of adhesion- related complications of $2350 following open surgery and $970 after laparoscopy within the first 5 years after surgery. Adhesion barriers reduced these costs by between $328 and $680 after open surgery; in laparoscopy the costs ranged from $63 in savings to $82 in expenses (Ten Broek et al., 2016). Another study (Hernandez et al., 2019) has shown that patient comorbidity was similar across different Emergency General Surgery (EGS) disease severity grades system as per the American Association for the Surgery of Trauma (AAST). AAST-EGS grade I refers to partial small bowel obstruction (SBO), grade II is complete SBO with viable, not compromised bowel, grade III means complete SBO with compromised but viable bowel, grade IV refers to complete SBO with non- viable bowel or perforation with localized spillage, and grade V means small bowel perforation with diffuse peritoneal contamination. In this study, median direct and indirect costs of adhesion increased by 1.4-fold, 1.6-fold, and 4.3-fold for AAST-EGS grade II, III, and IV, respectively, relative to grade I (Hernandez et al., 2019).

More research needs to be conducted to allow for cost-effective and personalised use of adhesion barriers based on validated risk indicators. A study estimating the cost effectiveness of the adhesion barrier Interceed (Roy et al., 2015) showed that the material cost of using the adhesion barrier was offset by a factor of almost 5 by the reduction in length of stay, fewer adhesion-related readmissions, and operating room cost.

### New developments in the area of adhesion prevention

It is important to note that due to the enormous complexity of the pathophysiology of adhesion formation, ineffective or difficult-to-use products, or safety issues, it is currently not possible to consistently treat or prevent post-operative adhesion formation (Fatehi Hassanabad et al., 2021). Future research needs to widen its glance to not only focus on isolated processes such as inflammation or the coagulation cascade but must address their crosstalk in order to better understand what causes the physiologically necessary wound healing process to become pathogenic. Better understanding of the underlying processes will support the ongoing research on personalised drugs that may ultimately prevent or even heal adhesions in a safe, effective, and precise manner. Current research also evaluates the combined use of mechanical barriers, adjuvants such as anti- inflammatory agents or hormones, and targeted gene therapy (Capella-Monsonís et al., 2019). The effect of stem cell therapy on tissue repair has also been investigated in preclinical models and clinical trials. These trials investigated menstrual, bone marrow, umbilical cord, and adipose tissue- derived stem cells in the prevention of intrauterine adhesions (Asherman syndrome) and demonstrated their safety and effectiveness in resumption of menstruation, fertility outcomes, and endometrial regeneration (Benor et al., 2020).

## Conclusion

Post-surgical adhesions are a frequently occurring adverse consequence of abdominopelvic surgery. They arise from pathological imbalances in the wound healing process, whose complexity is not yet fully elucidated. Adhesions represent a large burden on patients and on healthcare resources. Efforts need to be made to raise awareness about their importance and adequate preventive measures amongst surgeons and patients. Furthermore, there still is a need to prospectively validate the existing adhesion score. Such a score might help surgeons to decide when an adjunct tool is needed. Health economic evaluations of existing preventive measures are required to ensure broad access to cost-effective anti-adhesive agents. In the long-term, better understanding of the processes underlying the formation of adhesions will allow for their targeted prevention and therapy.
